# Free Glycogen in Vaginal Fluids Is Associated with *Lactobacillus* Colonization and Low Vaginal pH

**DOI:** 10.1371/journal.pone.0102467

**Published:** 2014-07-17

**Authors:** Paria Mirmonsef, Anna L. Hotton, Douglas Gilbert, Derick Burgad, Alan Landay, Kathleen M. Weber, Mardge Cohen, Jacques Ravel, Gregory T. Spear

**Affiliations:** 1 Department of Immunology/Microbiology, Rush University, Chicago, Illinois, United States of America; 2 The CORE Center, Cook County Health & Hospital System, Chicago, Illinois, United States of America; 3 Institute for Genome Sciences, University of Maryland School of Medicine, Baltimore, Maryland, United States of America; Fred Hutchinson Cancer Center, United States of America

## Abstract

**Objective:**

*Lactobacillus* dominates the lower genital tract microbiota of many women, producing a low vaginal pH, and is important for healthy pregnancy outcomes and protection against several sexually transmitted pathogens. Yet, factors that promote *Lactobacillus* remain poorly understood. We hypothesized that the amount of free glycogen in the lumen of the lower genital tract is an important determinant of *Lactobacillus* colonization and a low vaginal pH.

**Methods:**

Free glycogen in lavage samples was quantified. Pyrosequencing of the 16S rRNA gene was used to identify microbiota from 21 African American women collected over 8–11 years.

**Results:**

Free glycogen levels varied greatly between women and even in the same woman. Samples with the highest free glycogen had a corresponding median genital pH that was significantly lower (pH 4.4) than those with low glycogen (pH 5.8; p<0.001). The fraction of the microbiota consisting of *Lactobacillus* was highest in samples with high glycogen versus those with low glycogen (median = 0.97 vs. 0.05, p<0.001). In multivariable analysis, having 1 vs. 0 male sexual partner in the past 6 months was negatively associated, while BMI ≥30 was positively associated with glycogen. High concentrations of glycogen corresponded to higher levels of *L. crispatus* and *L. jensenii,* but not *L. iners*.

**Conclusion:**

These findings show that free glycogen in genital fluid is associated with a genital microbiota dominated by *Lactobacillus,* suggesting glycogen is important for maintaining genital health. Treatments aimed at increasing genital free glycogen might impact *Lactobacillus* colonization.

## Introduction

In many women, a healthy lower genital microbiota consists predominantly of *Lactobacillus* species such as *L. crispatus, L. jensenii, L. gasseri* or *L. iners* [1]–[4]. A key feature of colonization by lactobacilli is the relatively low pH in the genital tract (pH≤4.5), due to the ability of these bacteria to produce large amounts of lactic acid [5], [6]. This low pH helps protect against colonization by other microbes [5]–[8]. *Lactobacillus* species may also promote vaginal health by producing other antimicrobial compounds such as bacteriocins [9], [10].

The lack of vaginal colonization with *Lactobacillus* is associated with the development of conditions such as bacterial vaginosis (BV), often associated with adverse medical consequences in women [11]–[13]. Women with a genital microbiota dominated by lactobacilli are at significantly lower risk of acquisition of HIV, HSV-2, and several other sexually transmitted pathogens, including *Neisseria gonorrhoeae* and *Chlamydia trachomatis* [14]–[18]. In HIV infected women, those with higher genital levels of lactobacilli shed less HIV virus in their vaginal fluids [19], [20] and are at lower risk of transmitting HIV to their partner [21]. Colonization with lactobacilli is also associated with lower risk of pelvic inflammatory disease and pregnancy-related complications, including pre-term labor [4], [22]–[25]. *Lactobacillus* therefore plays an important role in maintaining health.

Different species of *Lactobacillus* offer varying degrees of protection against vaginal pathogens. Colonization with *L. crispatus* and *L. jensenii* is considered to be most beneficial while *L. iners* and *L. gasseri* are thought to be less protective [1], [26]–[29]. Identifying the different species of *Lactobacillus* and factors that help their colonization is important for understanding susceptibility to pathogens and preventing adverse outcomes in pregnancy.

It has long been recognized that vaginal *Lactobacillus* levels and vaginal pH vary over the life course of women [30]–[32]. At the onset of puberty, the female lower genital tract typically becomes colonized predominately with *Lactobacillus* and the vaginal pH decreases. These changes coincide with an increase in circulating estrogen and a rise in glycogen deposits in vaginal epithelial cells [30]. This association between colonization with *Lactobacillus* and deposition of intraepithelial glycogen has led to the hypothesis that glycogen serves as an important energy source for lactobacilli and their ability to colonize and produce lactic acid in the female lower genital tract [12], [30].

However, questions remain as to how glycogen promotes *Lactobacillus* colonization in the female lower genital tract. It is not known how much glycogen is available for *Lactobacillus* utilization in the vaginal milieu. While the glycogen-containing vaginal epithelial layer can vary in thickness depending on hormonal concentrations [30], [31], [33], [34], it is not clear how this would affect free glycogen levels in the vaginal lumen, where lactobacilli grow. Moreover, several studies have shown that most isolates of *Lactobacillus -*at least *in vitro*-do not directly utilize glycogen [35]–[38], even when the glycogen was isolated from the genital tract of women [37].

In this study, we investigated the hypothesis that free glycogen (i.e. soluble glycogen in genital fluid that was not cell-associated) correlates with *Lactobacillus* colonization. We therefore measured free glycogen in genital fluids and assessed its relationship with *Lactobacillus* levels, different *Lactobacillus* species, and vaginal pH.

## Materials and Methods

### Ethics Statement

The study was approved by The Rush University Medical Center and The Cook County Health & Hospital System Institutional Review Boards (IRBs). Written informed consent was obtained from all participants according to protocols approved by The Rush University Medical Center and The Cook County Health & Hospital System IRBs.

### Participants and Sample Acquisition

All subjects included in this analysis were HIV seronegative African American women from the Chicago site of the Women’s Interagency HIV Study (WIHS), who reported similar socio-demographic and behavioral risk factors to the HIV infected women enrolled in the Chicago WIHS, and were selected as matched controls for a longitudinal study of the effect of HIV infection on vaginal microbiota (manuscript in preparation). Exclusion criteria for the larger longitudinal study included having gonorrhea, chlamydia, syphilis, or trichomoniasis at the index study visit, being post-menopausal at any time during the study, and any reported exchange of sex for money or drugs during the study period. A detailed structured psychosocial and behavioral interview, physical and gynecologic examinations, along with both blood and gynecologic sampling and testing were performed at the time of sample collection. The presence of *Trichomonas vaginalis*, *Candida albicans*, and HSV were determined by wet mount, KOH preparation, and visual exam for lesions, respectively. Bacterial vaginosis (BV) was determined by Amsel’s criteria, defined as having all three of the following factors present: vaginal pH>4.5, clue cells, and amine odor [39]. The pH of vaginal secretions was determined prior to cervico-vaginal lavage (CVL) collection, by adding genital fluid to indicator strips with a pH range of 4–7 (ColorpHast indicator strips, MCB Reagents, Gibbstown, NJ). Genital tract fluids were then collected by CVL, performed by irrigation of the cervix with 10 ml of non-bacteriostatic sterile saline, followed by aspiration from the posterior fornix. Genital fluid samples were kept cold until processing, which occurred within 6 hours of collection. Samples were gently vortexed to evenly distribute cells before storage at −80°C.

### DNA Isolation, PCR Amplification, And Pyrosequencing of Barcoded 16S rRNA Gene V1-V2 Regions Amplicons

Bacteria from genital fluid samples were pelleted by centrifugation at 13200 rpm for 25 min. Supernatants were stored at −70°C for glycogen measurement. Bacteria pellets were re-suspended in 200 µl nuclease free water (Qiagen, Valencia, CA). DNA isolation was performed using FastDNA SPIN kit (MP Biomedicals, Solon, OH). Bacterial pellets were added to the Lysing Matrix A tube and beads agitated for 40 seconds at 6 m/s (FastPrep-24, MP Biomedicals, Santa Ana, CA). Final elution volume was 100 µl. DNA concentration was determined by NanoDrop 2000 spectrophotometer. All procedures were done at room temperature.

Pyrosequencing of barcoded 16S rRNA gene V1-V2 region amplicons was performed as described previously [40]. Briefly, barcoded sets of domain-level primers (*i.e.* 27F and 338R) containing an eight-base barcode were used to amplify the V1 and V2 hypervariable regions of bacterial 16S rRNA genes from genomic DNA extracts. The primers were as follows: 27F-5′-GCCTTGCCAGCCCGCTCAGTC**AGAGTTTGATCCTGGCTCAG**-3′ and 338R-5′-GCCTCCCTCGCGC- CATCAGNNNNNNNNCAT**GCTGCCTCCCGTAGGAGT**-3′, where the underlined sequences are the 454 Life Sciences FLX sequencing primers B and A in 27F and 338R, respectively, and the bold letters denote the universal 16S rRNA primers 27F and 338R. The 8-bp barcode within primer 338R is denoted by 8 Ns. Barcoded amplicons were pooled in equimolar concentration, and sequenced on a Roche/454 FLX instrument (Branford, CT), using titanium technology. Component of the QIIME software (version 1.6.0) [41] was used to bin the sequences based on their barcode, trim the primers and barcodes, remove sequences with homopolymeric runs longer than 8 bp, with any ambiguous base calls and/or shorter than 150 bp. In addition, detection of chimeric sequences was performed using the UCHIME component of UCLUST [42] and chimeric sequences were removed. Bacteria taxonomic assignments were performed using a combination of the Naïve Bayesian Classifier [43] trained on version 10 of the RDP database [43] and pplacer [40], [44]. Each sample was run once since our past study indicates that duplicates of the PCR amplification and pyrosequencing steps have relatively low variability [40].

### Glycogen Measurement

Free glycogen in genital fluid samples was measured fluorometrically using the Glycogen Assay Kit (BioVision, Milpitas, CA). Samples were thawed on ice and vortexed for 10 seconds. Genital fluid or saline (serving as blank) was added into 4 wells (10 µl/well) in a 96-well plate. The volume was adjusted to 50 µl with hydrolysis buffer, with or without the hydrolysis enzyme (2 wells each). The wells without enzyme constituted the glucose background. Glucose background was subtracted from final readings to determine glycogen concentration. The Relative Fluorescence Units (RFU) were measured using a BioTek Synergy HT plate reader (BioTek Instruments, Inc, Winooski, VT).

### Variable Classifications And Statistical Analysis

Explanatory variables for analysis included age in years, highest level of education completed (less than high school, completed high school, college or graduate degree), and annually, current smoking status (yes vs. no), body mass index (BMI), number of male sex partners in the past 6 months, oral contraceptive use (yes vs. no), parity (0–1 vs. ≥2), and history of tubal ligation (yes vs. no). BMI was collected as a continuous variable and analyzed according to standard categories as normal weight (18.5–24.9); overweight (25.0–29.9), and obese (≥30). Total number of male sex partners was collected as a continuous variable; however, across all study visits, 26% reported 0 sex partners and 59% reported 1 sex partner, so number of sex partners was analyzed as a categorical exposure as 0, 1, or ≥2 partners. *Lactobacillus* relative abundance obtained from 16S rRNA gene vaginal microbiota analysis was bimodally distributed and dichotomized for analysis as ≥85% vs. <85%; this categorization demonstrated content validity with bacterial vaginosis (BV) as determined by Amsel’s criteria, whereby BV prevalence was 1.4% (1/69) for women with *Lactobacillus* relative abundance ≥85% compared to 45.9% (50/109) for women with *Lactobacillus* relative abundance <85%. The distribution of glycogen demonstrated significant skewness which was not improved by any transformations (including square root, logarithmic, and exponential transformation) or standardization. Because there are no clinically relevant cutpoints for free vaginal glycogen, glycogen was analyzed as a binary variable dichotomized about the median (0.026 µg/µl) and categorically using cutpoints according quartiles of the distribution (≤0.009; 0.009–0.026; 0.026–0.064; and 0.064–0.425 µg/µl).

Across all annual study visits, there were 189 valid glycogen and 178 valid microbiome measurements. The final analytic sample consisted of 177 observations with non-missing values for both glycogen and microbiome. The distributions of non-normally distributed continuous variables (*Lactobacillus* sp. relative abundance, and vaginal pH) were analyzed by quartiles of glycogen concentration using an equivalent to the Kruskal-Wallis test with adjustment for clustering among repeated measures by estimating the cluster-adjusted Somers’ D parameter for each quartile of glycogen and testing the null hypothesis of zero association across all quartiles [45]. To account for correlation between repeated measurements on individuals over time, we used generalized estimating equations (GEEs) with an exchangeable correlation structure to fit complementary log-log regression models, an alternative to the logistic regression model for binary responses with skewed distributions [46]. GEE models utilize robust variance estimation (sandwich estimator) to adjust standard errors for non-independence of repeated measures. Exponentiated coefficients yield odds ratios with the same interpretation as those from the logit model. To identify factors that may confound the association between *Lactobacillus* relative abundance and glycogen and to examine factors associated with glycogen, we conducted separate analyses for glycogen and *Lactobacillus* relative abundance as outcome variables. Variables with p<0.2 in univariable analysis and those that were considered important based on *a priori* hypotheses were entered in multivariable models; stepwise methods were used to select predictors for the final multivariable models. Variables that were statistically significant at p<0.05 in multivariable analysis or that were considered conceptually important were retained in the final models. An indicator for time was included in all models to adjust for variation over time in glycogen and *Lactobacillus* relative abundance. Data were analyzed using STATA version 13.1 (STATA Corp, College Station, TX).

## Results

### Distribution of Relative Abundance of *Lactobacillus* by Glycogen Concentration

Vaginal fluid samples were collected annually by cervicovaginal lavage from 21 African-American HIV-seronegative women over a period of 8–11 years with an average of 8.8 samples per subject. Index visits took place in 1994–1995 for 8/21 (38%) subjects and 2001–2002 for 13/21 (62%) subjects. Subjects’ characteristics at the index study visit are summarized in Table 1. The median age at index visit was 28.5 (range 18.6–40.0) years. At the index visit, 29% of individuals were overweight and 57% were obese; 66.7% of subjects were current smokers. Of the 177 samples that were analyzed, 11.3% of the samples were collected when women tested positive for the presence of *T. vaginalis*, while 2.8% were from women positive for *C. albicans*, and 28.8% were from women that had clinically-diagnosed BV (Amsel’s criteria). None of the women had identifiable lesions requiring HSV culture. The median vaginal pH was 5.3 (range: 4.0–7.0).

**Table 1:** Subjects’ Characteristics at Index Visit, N = 21.

	n (%)
**Age in years, median (range)**	28.5 (18–40)
**Educational attainment**	
Less than high school	6 (28.6)
High school graduate	8 (38.1)
Some college or above	7 (33.3)
**BMI**	
18.5–24.9 (normal weight)	3 (14.3)
25.0–29.9 (overweight)	6 (28.6)
≥30 (obese)	12 (57.1)
**Parity**	
0–1	7 (33.3)
≥2	14 (66.7)
**Tubal ligation ever**	2 (9.5)
**Oral Contraceptive use in past 6 months**	3 (14.3)
**Current smoking**	14 (66.7)
**Number of male sex partners in past 6 months**	
0	3 (14.3)
1	12 (57.1)
≥2	6 (28.6)
**Clinically detected BV by Amsel’s criteria**	5 (23.8)
**pH, median (range)**	5.0 (4.0–7.0)

**Figure 1:**
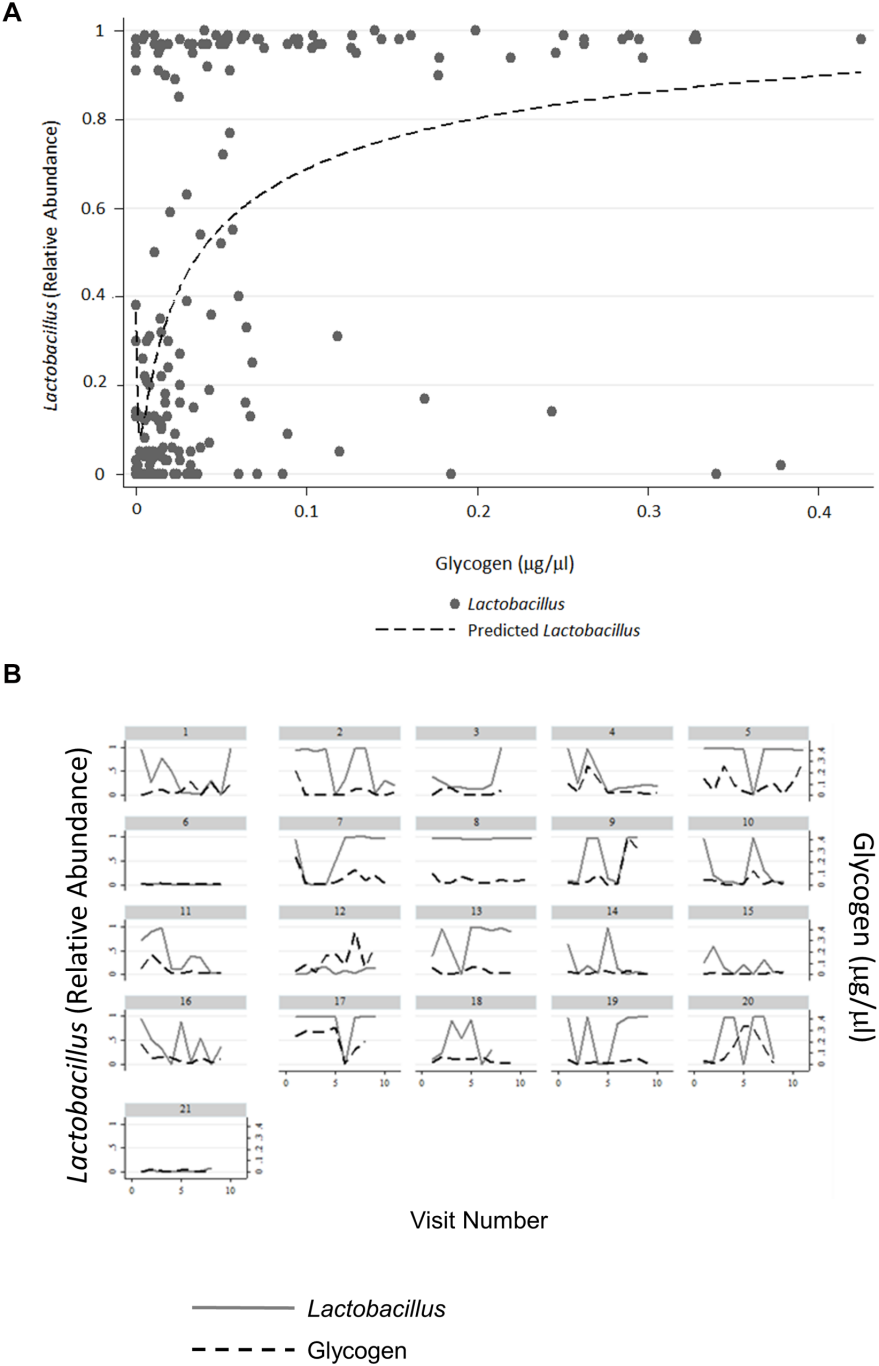
Distribution of Relative Abundance of *Lactobacillus* by Glycogen Concentration. Glycogen levels were measured in vaginal fluids collected annually from 21 women over 8–11 years. (A) The final analytic sample consisted of 177 observations with non-missing values for both glycogen and *Lactobacillus*. The distribution of *Lactobacillus* relative abundance is summarized by glycogen as a continuous variable. (B) The relative abundance of *Lactobacillus* and concentration of glycogen over time by participant. Left Y axis is relative abundance of *Lactobacillus*; right Y axis is glycogen concentration (µg/µl). X-axis is annual visit number.

The amount of free glycogen for each of the 21 women across all visits was measured and ranged from 0 to 0.425 µg/µl with a median of 0.026 µg/µl (Fig. 1A). When examined at the individual level, some women had consistently low levels of free glycogen (Fig. 1B, subjects 6, 8, and 21 for example), while others maintained higher levels of glycogen over time (e.g. subjects 12, and 20). In most women, however, glycogen concentrations fluctuated between relatively low and high levels at different visits (Fig. 1B, e.g. subjects 1 and 12).

The *Lactobacillus* relative abundance in vaginal samples was bimodal, ranging from 0–100%; the mean and median were 45.9% and 28.3%, respectively (Fig. 1A). There was a strong positive association between *Lactobacillus* relative abundance and free glycogen (Spearman r = 0.48, p<0.001). Again, there was a significant variation in *Lactobacillus* relative abundance between women and within women over time (Fig. 1B, e.g. subjects 8 and 16). The relative abundance of both *L. crispatus* and *L. iners* also fluctuated considerably among women and in each woman at different visits (Figure S1). We found there was no significant association between glucose levels and *Lactobacillus* relative abundance in vaginal fluids (Spearman r = 0.07, p = 0.37; Figure S2A). Moreover, no significant correlation was observed between free glycogen levels and glucose in the vaginal fluids (Figure S2B).

We next determined which factors might contribute to the variability in glycogen levels measured in vaginal fluids. In univariable analysis, we found that having a BMI ≥30 and a history of tubal ligation were associated with increased odds of having glycogen >0.026 µg/µl. Moreover, current smoking, and having 1 vs. 0 male sexual partners in the past 6 months were associated with lower glycogen levels. In contrast, age, education, parity, and oral contraceptive use were not associated with glycogen level, and there was no change over time in the proportion of patients with high glycogen at the population level (Table 2).

**Table 2:** Factors Associated with High Glycogen (>0.026 µg/µl), N = 177 visits.

	Univariable OR*(95% CI)	p-value	MultivariableOR* (95% CI)	p-value
**Age in years**	1.02 (0.97–1.06)	0.461	–	–
**Education**				
Less than high school	1.0 (Ref.)	–	–	–
High school graduate	1.67 (0.78–3.56)	0.187	–	–
Some college or above	1.18 (0.56–2.52)	0.660	–	–
**BMI**				
18.5–24.9	1.0 (Ref.)	–	–	–
25.0–29.9	1.57 (0.79–3.11)	0.196	1.50 (0.69–3.24)	0.307
≥30	2.10 (1.02–4.32)	0.045	2.21 (0.98–5.02)	0.057
**Parity≥2 vs. 0–1**	2.10 (0.86–5.13)	0.106	–	–
**Tubal ligation ever**	1.75 (1.07–2.87)	0.025	–	–
**Oral contraceptive use**	1.31 (0.66–2.60)	0.444	–	–
**Current smoking**	0.53 (0.32–0.89)	0.016	–	–
**Male sex partners, past 6 months**				
0	1.0 (Ref.)	–	1.0 (Ref)	–
1	0.48 (0.28–0.85)	0.011	0.46 (0.27–0.80)	0.006
≥2	0.76 (0.41–1.41)	0.384	0.77 (0.38–1.55)	0.259
**Visit**	0.98 (0.92–1.04)	0.536	0.97 (0.90–1.04)	0.357

OR = Odds Ratio; CI = Confidence Interval; Ref. indicates reference category.

*Odds Ratios generated from complementary log-log function.

Multivariable model is adjusted for variables for which estimates are presented.

Because glycogen expression by vaginal epithelial cells is thought to be influenced by changes in estrogen levels [47], we examined whether free glycogen levels varied according to quartiles of the self-reported menstrual cycle, but found they did not significantly change at different stages of the cycle (Figure S3).

In multivariable analysis, having 1 vs. 0 male sex partners in the past 6 months was associated with a lower odds of high glycogen (aOR 0.46; 95% CI 0.27–0.80); the association between having ≥2 vs. 0 male partners was not statistically significant (aOR 0.77; 0.38–1.55). Having a BMI ≥30 was marginally associated with increased odds of high glycogen (aOR 2.21; 95% CI 0.98–5.02; Table 2).

### High free glycogen is associated with a vaginal microbiota dominated by *Lactobacillus* and a low vaginal pH

**Figure 2:**
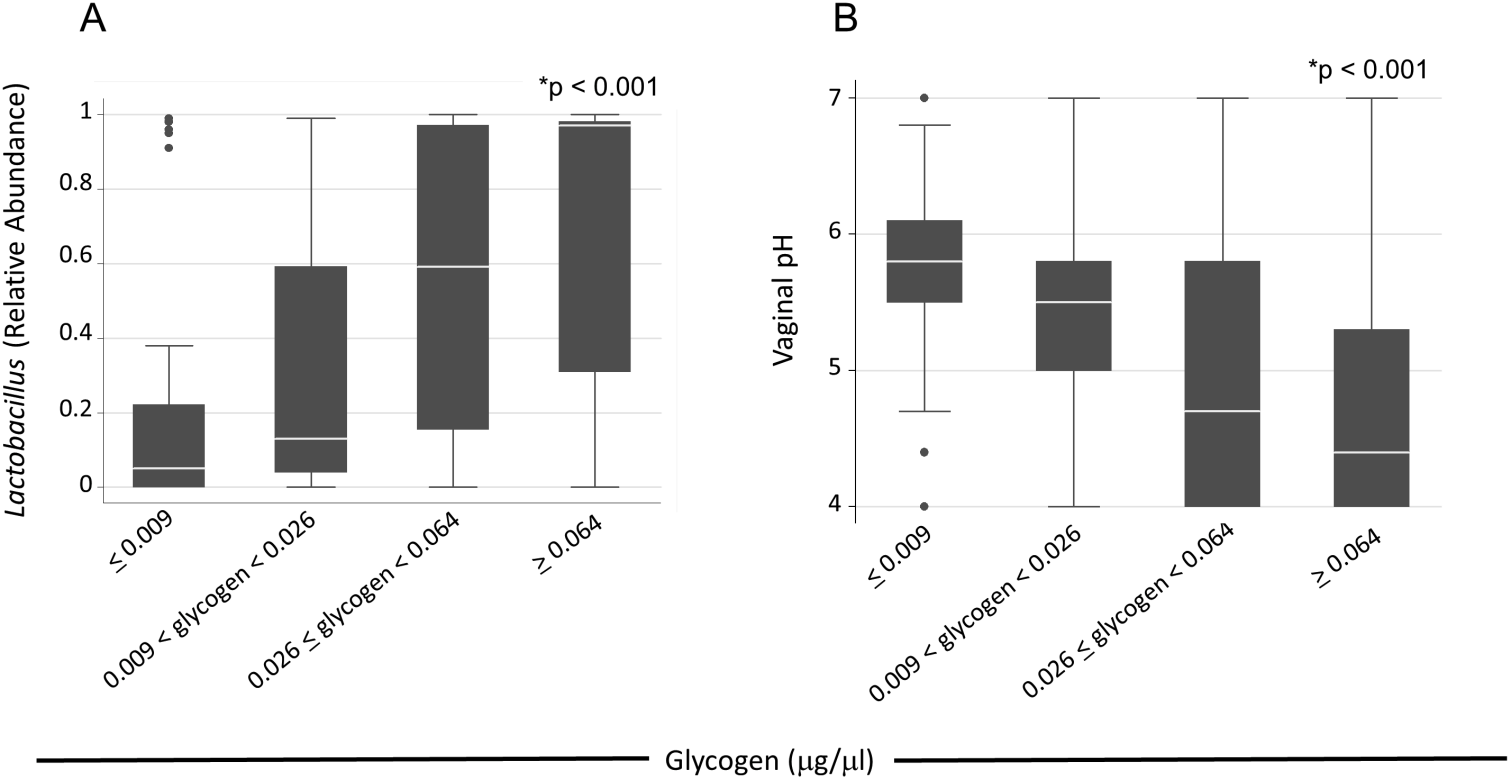
High levels of free glycogen are associated with a vaginal microbiota dominated by *Lactobacillus* species and a low vaginal pH. The final analytic sample consisted of 177 observations with non-missing values for both glycogen and *Lactobacillus*. Free glycogen was analyzed as a binary variable dichotomized about the median and categorically using cutpoints according quartiles of the distribution, as indicated. The distribution of *Lactobacillus* relative abundance (A) and vaginal pH (B) are summarized by glycogen as a continuous variable by glycogen quartiles. *p-value is estimated by extension of the Kruskal-Wallis test with adjustment for clustering among repeated measurements.

Because a microbiota predominantly colonized by *Lactobacillus* is associated with reduced likelihood of contracting/transmission sexually transmitted infections (STIs) and improved pregnancy outcomes [13], pyrosequencing of the 16S rRNA gene was used to determine the fraction of bacterial microbiota that was *Lactobacillus*. When analyzed according to quartiles of glycogen concentration, *Lactobacillus* was most abundant in samples with the highest level of free glycogen [median relative abundance = 0.97 (IQR 0.31–0.98), Fig. 2A], and was significantly lower in samples with the lowest amounts of free glycogen [median relative abundance = 0.05 (IQR 0.00–0.22), p<0.001].

Since a low vaginal pH (≤4.5) is thought to be a key mechanism for the health benefits associated with *Lactobacillus* colonization [5], [6], [13], [48], we examined vaginal pH by quartiles of glycogen concentration. Vaginal pH decreased with increasing glycogen concentration, from median 5.8 (IQR 5.5–6.1) among those with the lowest glycogen concentration, to median 4.4 (IQR 4.0–5.3) in samples with the highest glycogen concentration (p<0.001; Fig. 2B). As expected, vaginal pH was significantly lower in samples having a *Lactobacillus* relative abundance of ≥85% (Figure S4).

While 20 out of 177 samples were positive for *Trichomonas*, the *Trichomonas*+ samples had significantly lower levels of *Lactobacillus* (median = 0.14) compared to those without *Trichomonas* (median = 0.34, p = 0.02). However, glycogen was not different in *Trichomonas*+ vs. *Trichomonas*– samples.

To ascertain what factors were associated with high *Lactobacillus* relative abundance (≥85%), and hence low prevalence of clinically diagnosed BV (1.4%; see Methods), univariable analyses were performed. In addition to higher levels of glycogen, education, BMI 25–29.9, and oral contraceptive use were all found to be significantly associated with a greater odds of *Lactobacillus* relative abundance ≥85% (Table 3). Conversely, having more recent male sex partners was associated with lower odds of high *Lactobacillus* relative abundance. Time, age, parity, history of tubal ligation, and current smoking were not associated with *Lactobacillus* relative abundance. In multivariable analysis, which controlled for education, BMI, and oral contraceptive use, there was a strong positive association between glycogen levels and *Lactobacillus* relative abundance (Table 3).

**Table 3:** Factors Associated with *Lactobacillus* ≥85%, N = 177 visits.

	Univariable OR* (95% CI)	p-value	Multivariable OR* (95% CI)	p-value
**Glycogen, µg/µl**				
≤0.009	1.0 (Ref.)	–	1.0 (Ref)	–
<0.026	1.68 (0.63–4.48)	0.298	2.57 (1.07–6.17)	0.035
<0.064	3.36 (1.24–9.16)	0.018	5.87 (1.90–18.1)	0.002
0.064–0.425	8.15 (2.86–23.2)	<0.001	25.2 (7.08–89.8)	<0.001
**Age at visit**	1.01 (0.94–1.08)	0.873	–	–
**Education**				
Less than high school	1.0 (Ref.)	–	1.0 (Ref)	–
High school graduate	1.20 (0.38–3.77)	0.754	1.92 (0.26–14.3)	0.523
Some college or above	3.39 (1.24–9.29)	0.017	8.12 (1.09–60.4)	0.041
**BMI**				
18.5–24.9	1.0 (Ref.)	–	1.0 (Ref)	–
25.0–29.9	2.36 (1.19–4.67)	0.014	3.11 (1.31–7.37)	0.010
≥30	1.30 (0.53–3.23)	0.565	0.90 (0.32–2.48)	0.834
**Parity≥2 vs. 0–1**	1.20 (0.40–3.61)	0.751	–	–
**Tubal ligation**	1.75 (0.78–3.94)	0.173	–	–
**Oral contraceptive use**	3.05 (1.48–6.29)	0.002	12.8 (4.31–38.2)	<0.001
**Current smoking**	0.56 (0.26–1.21)	0.139	–	–
**Male sex partners, past 6 months**				
0	1.0 (Ref.)	–	–	–
1	0.47 (0.27–0.82)	0.008	–	–
≥2	0.51(0.24–1.08)	0.078	–	–
**Visit**	0.99 (0.90–1.10)	0.883	1.01 (0.90–1.12)	0.904

OR = Odds Ratio; CI = Confidence Interval; Ref. indicates reference category.

*Odds Ratios generated from complementary log-log function.

Multivariable model is adjusted for variables for which estimates are presented.

### Relationship of glycogen to different species of *Lactobacillus*

**Figure 3:**
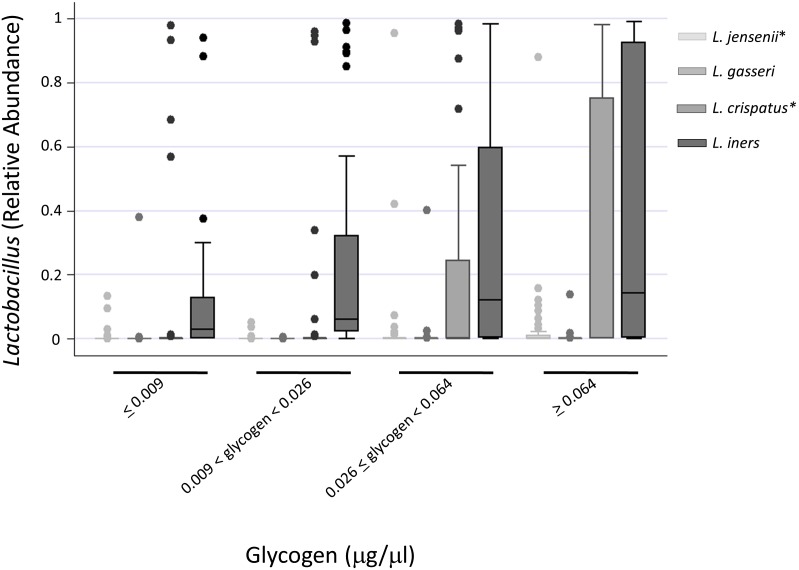
Relative Abundance of *Lactobacillus* Species by Category of Glycogen Concentration. Free glycogen was analyzed as a binary variable dichotomized about the median and categorically using cutpoints according quartiles of the distribution, as indicated. The distribution of *Lactobacillus* species relative abundance is summarized by glycogen as a continuous variable by glycogen quartiles. Shaded portions represent interquartile range, horizontal bars represent medians, and whiskers represent 95% confidence intervals. *p<0.05 using extension of the Kruskal-Wallis test with adjustment for clustering among repeated measurements.

Of the main species of *Lactobacillus* known to colonize the female genital tract, *L. crispatus* and *L. jensenii* are regarded as most protective while *L. iners* and *L. gasseri* may be less protective against vaginal pathogens [13]. When the relative abundance of these *Lactobacillus* species were assessed in all samples, *L. iners* was found to be the most predominant species, followed by *L. crispatus* (Fig. 3).

Interestingly, the relative abundance of *L. iners* did not vary significantly across glycogen categories, increasing from a median 0.03 (IQR 0.00–0.129) at lowest free glycogen concentration to 0.142 (IQR 0.002–0.926) at highest glycogen concentration (p = 0.125; Fig. 3). In contrast, *L. crispatus* relative abundance significantly increased from median 0.00 (IQR 0.00–0.001) to 0.002 (IQR 0.00–0.753) at the lowest and highest concentrations of free glycogen, respectively (p = 0.011). *L. jensenii* and *L. gasseri* each comprised <2% of all microbiota, but only *L. jensenii* levels varied significantly by concentration of free glycogen (Figure 3).

## Discussion

By measuring glycogen deposits in vaginal epithelium, earlier investigators noted the absence of glycogen in pre-pubescent girls, the appearance of glycogen in post-pubescent women, and that post-menopausal women had lower levels of intraepithelial glycogen [30], [31]. The appearance of glycogen was concurrent with vaginal colonization by *Lactobacillus* and a decrease in vaginal pH [30], [31]. These observations led to the hypothesis that lactobacilli could use glycogen as an energy source to grow and to produce large amounts of lactic acid in the lumen, which is key for the ability of lactobacilli to promote vaginal health [5], [6], [12], [48].

We advanced these investigations by quantifying the amount free glycogen in genital fluids that would be available for utilization by *Lactobacillus* species. We then assessed the relationship between glycogen and *Lactobacillus* relative abundance using a molecular method to measure the proportion of genital bacteria that consisted of *Lactobacillus* since culture methods do not accurately report genital bacteria abundance. We observed that free glycogen levels in vaginal fluids are strongly associated with both the proportion of genital microbiota that is comprised of *Lactobacillus* species and a low vaginal pH. These observations are potentially of medical importance since lactobacilli in the female lower genital tract have a major impact on vaginal health, both in resistance STIs and in preventing pregnancy complications [13], [48]. Thus, our data suggest that the amount of glycogen in genital secretions can significantly impact *Lactobacillus* species colonization.

Surprisingly, we found a large variability in free glycogen levels among women, and even in the same woman at different times. The samples were taken at one-year intervals and it is not clear what level of variation occurs over a shorter time frame. This variation is potentially important because such fluctuations in available glycogen may profoundly affect *Lactobacillus* species colonization. We speculate that a prolonged and/or consistent period of low free glycogen availability may put a woman at risk of bacterial vaginosis, as well as acquisition and potentially transmission of STIs [49].

To investigate the cause of the variability, we compared glycogen with menstrual cycle phase using self-reported date of last menses, since changes in glycogen in the vaginal epithelium during different stages of development (pre-puberty, puberty, menopause) have been known for some time; thus implicating estrogen in inducing glycogen expression [47]. Interestingly, the observed variability in free glycogen levels did not appear to be due to hormonal changes as the different stages of menstrual cycle, reported as days since last menstrual period were not significantly associated with high levels of free glycogen (Figure 3).

It is possible that misreporting of last menses could have obscured this relationship. However, Gregoire et al. reported that glycogen, quantified in hydrolyzed vaginal biopsies, was independent of hormonal changes associated with menstrual cycle [50], suggesting a disconnect between estrogen and glycogen. Moreover, significant levels of glycogen have been detected in cells from vaginal smears of women who have undergone bilateral ovariectomy [51], as well as in one study of post-menopausal women [52]. On the other hand, administration of estrogens resulted in thickness of vaginal epithelium and an increase in both the number of glycogen-containing exfoliated vaginal cells and the amount of glycogen in the cells, as observed by microscopy [52], [53]. How estrogens produced by other sources (e.g. adipocytes [54]) can affect free glycogen levels remains to be determined. Investigators also observed a small, but significant, decrease in the thickness of epithelium that contained glycogen in vaginal biopsies as early as 3 months after Depoprovera administration [33]; however there were no significant differences in the thickness of glycogen-containing epithelium 12 months post Depoprovera administration [55].

These findings underscore the need for closely-spaced longitudinal measurements of estrogen to more definitively study the link between estrogen and free glycogen levels.

This may be difficult since over the menstrual cycle estrogen levels change in a few days by up to 10-fold [56] and any change in free glycogen might lag – by several days - the rapid rise (or fall) in estrogen. It is possible that estrogen could increase intraepithelial glycogen deposits on a per cell basis, and/or cause an increase in the thickness of epithelium that contains glycogen. However, these changes might have little effect on free glycogen levels in vaginal fluids. Instead, the rates at which glycogen-containing epithelial cells are shed, how much glycogen they contain, and how quickly they release glycogen might be more important contributors to free glycogen.

Another possible explanation for the variation in free glycogen observed between women could be that glycogen is differentially utilized or degraded by the varying types of microbiota, or by host-derived factors. We found that the highest amounts of free glycogen were found in women with a microbiota that is composed predominantly of *Lactobacillus* species. Therefore, if degradation explained the variation, this would suggest that *Lactobacillus* species cannot utilize or degrade free glycogen while the microbiota that is low in *Lactobacillus* species is able to utilize glycogen for its growth. However, this seems improbable given both the association between glycogen and *Lactobacillus* relative abundance noted during sexual development and that it is unlikely that women would produce a substance which would promote colonization by non-*Lactobacillus* microbiota, and not *Lactobacillus*. Thus, it is most likely that variation in glycogen is due to variation in production or release from the epithelium. Alternatively, as suggested by a number of *in vitro* studies, the lactobacilli’s ability to utilize glycogen may depend on the pH of environment [36], and/or the particular *Lactobacillus* species [36], [37]. A recent transciptomics analysis of *L. iners* showed that some glycosylases and a maltose transporter are expressed *in vivo* in this bacterium that could be involved in glycogen utilization [57].

Nutrition might be another factor that could affect free glycogen concentrations. The ingestion of carbohydrates is known to increase glycogen levels in liver and muscle cells [58], [59]. In our analysis we found that having a BMI ≥30 was associated with increased odds of having high glycogen, although this relationship was only marginally significant. In contrast, Willson et al. [52], found that increased carbohydrate intake in post-menopausal women did not significantly change the numbers of glycogen positive cells in vaginal smears.

In this study, while there was a wide range in free glycogen levels, the median level in the samples was about 0.026 µg/µl. Considering these samples were collected by lavage and thus diluted by approximately 30-fold [60], the actual median glycogen concentrations in undiluted genital fluids are estimated to be about 1 µg/µl. This corresponds to only about 0.1% carbohydrate in vaginal fluids. There may be some utilization and/or degradation of glycogen into smaller glucose-containing polymers in mucosal fluids. Therefore, the concentration of carbohydrates may be higher than 0.1%, correspondingly affecting the growth of *Lactobacillus* species in the vagina.

Similar to other studies [61]–[63], *L. iners* was found to be the most prevalent *Lactobacillus* species in this cohort (Figure 3). Among the different *Lactobacillus* species analyzed, *L. jensenii* and *L. crispatus* levels were significantly higher in samples with high concentrations of free glycogen (Figure 3). This is an important finding, because it suggests that colonization by these two *Lactobacillus* species -believed to be perhaps most protective [13]- are favored in presence of higher levels of glycogen.

Interestingly, however, *L. iners*’ relative abundance was not significantly higher in samples with the high levels of glycogen compared to those containing lower levels of glycogen, possibly due to the high variability between subjects (Figure 3). Similar to other reports [61], we also found samples containing more than 50% *L. iners* had a relatively higher pH (median = 4.4) than those with predominant *L. crispatus* (median = 4, p = 0.03). The ability to utilize glycogen by different *Lactobacillus* species could therefore impact the production of lactic acid by these bacteria, leading to observed differences in vaginal pH and conferring a different level of protection.

Vaginal colonization by *Lactobacillus* species and a low vaginal pH are shown to lower the risk of STIs, BV, and preterm labor. Therefore, understanding and supporting an environment that can promote colonization by *Lactobacillus* species is pivotal in maintaining vaginal health. The data presented herein identify free glycogen as an important factor that strongly associates with *Lactobacillus* relative abundance and a low vaginal pH in reproductive-aged women. We did not find the association between having ≥2 vs. 0 male sex partners to be statistically significant, possibly due to small sample size in the group reporting ≥2 sex partners.

This study has several limitations. Firstly, all of the women in this study were African American. Therefore, it is not certain that free glycogen levels measured here are representative of other racial/ethnic backgrounds. It will be important to assess free glycogen levels across racial groups, as it has been shown that microbiota composition can vary in women of different races [61]. Second, it should be noted that the relative abundance of bacteria in this study was assessed by using V1-V2 primers for 16S rRNA gene pyrosequencing, which may over- or under-represent some genera. *G. vaginalis*, a bacterium known to be a key member in the vagina, is likely under-represented in this study, and as a consequence *Atopobium vaginae* could be over-represented [64]; however, there are biases with any primer set. It will be interesting to compare how free glycogen levels are associated with vaginal microbiota as determined by other pyrosequencing methods.

Taken together, our data suggest that persistently low levels of free glycogen might increase a woman’s risk of adverse medical outcomes associated with low *Lactobacillus* levels. Furthermore, because different species of *Lactobacillus* provide different levels of protection, our studies have important implications on how the colonization of more beneficial lactobacilli may be achieved. These studies point to the importance of understanding the biological factors that affect levels of free glycogen and further indicate that designing interventions that increase and maintain high free glycogen might positively impact women’s health.

## Supporting Information

Figure S1 **The relative abundance of** ***Lactobacillus*** **species and concentration of glycogen over time by participant.** Left Y axis is relative abundance of *L. crispatus, L. iners, L. jensenii and L. gasseri*; right Y axis is glycogen concentration (µg/µl). X-axis is annual visit number.
(TIFF)

Figure S2 **Distribution of Glucose by** ***Lactobacillus*** **and Glycogen.** Glucose and glycogen levels were measured in vaginal fluids collected annually from 21 women over 8–11 years. The final analytic sample consisted of 177 observations with non-missing values for all variables. The distributions of *Lactobacillus* relative abundance (A) and glycogen (B) are summarized by glucose as a continuous variable.
(TIFF)

Figure S3 **Glycogen Concentration by Time Since Last Menstrual Period (LMP).** Shaded portions represent interquartile range, horizontal bars represent medians, and whiskers represent 95% confidence intervals. N = 149, excludes 6 visits where menopause or pregnancy was reported and 19 visits where LMP was >50 days.
(TIFF)

Figure S4 **pH by** ***Lactobacillus*** **Relative Abundance ≥85 vs. <85%.**
(TIFF)
