# Supplementary figures and images for: Free Glycogen in Vaginal Fluids Is Associated with Lactobacillus Colonization and Low Vaginal pH
Source: PLoS One. 2014 Jul 17;9(7):e102467. doi: 10.1371/journal.pone.0102467 (PMC4102502; doi:10.1371/journal.pone.0102467)

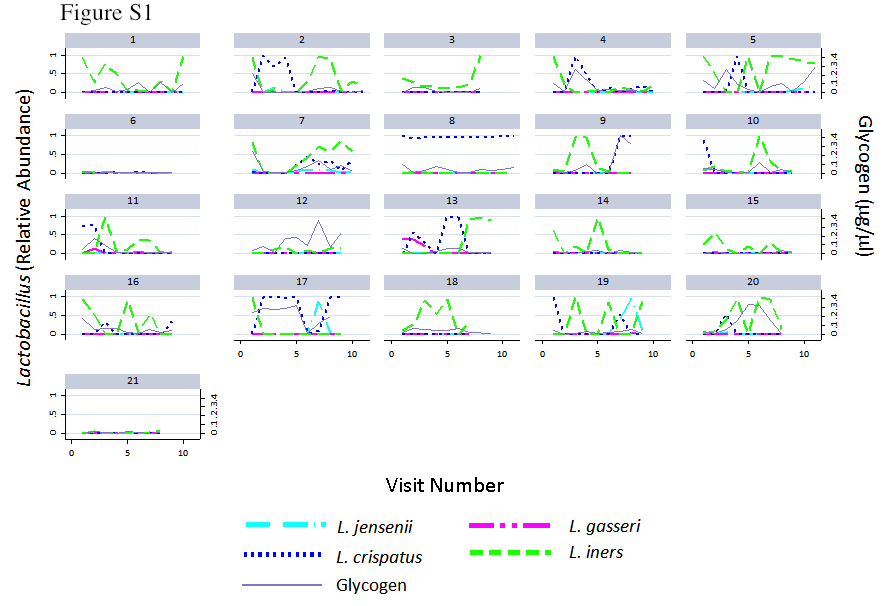

Supplement: Figure S1 — The relative abundance of Lactobacillus species and concentration of glycogen over time by participant. Left Y axis is relative abundance of L. crispatus, L. iners, L. jensenii and L. gasseri; right Y axis is glycogen concentration (µg/µl). X-axis is annual visit number. (TIFF) [file pone.0102467.s001.tiff]

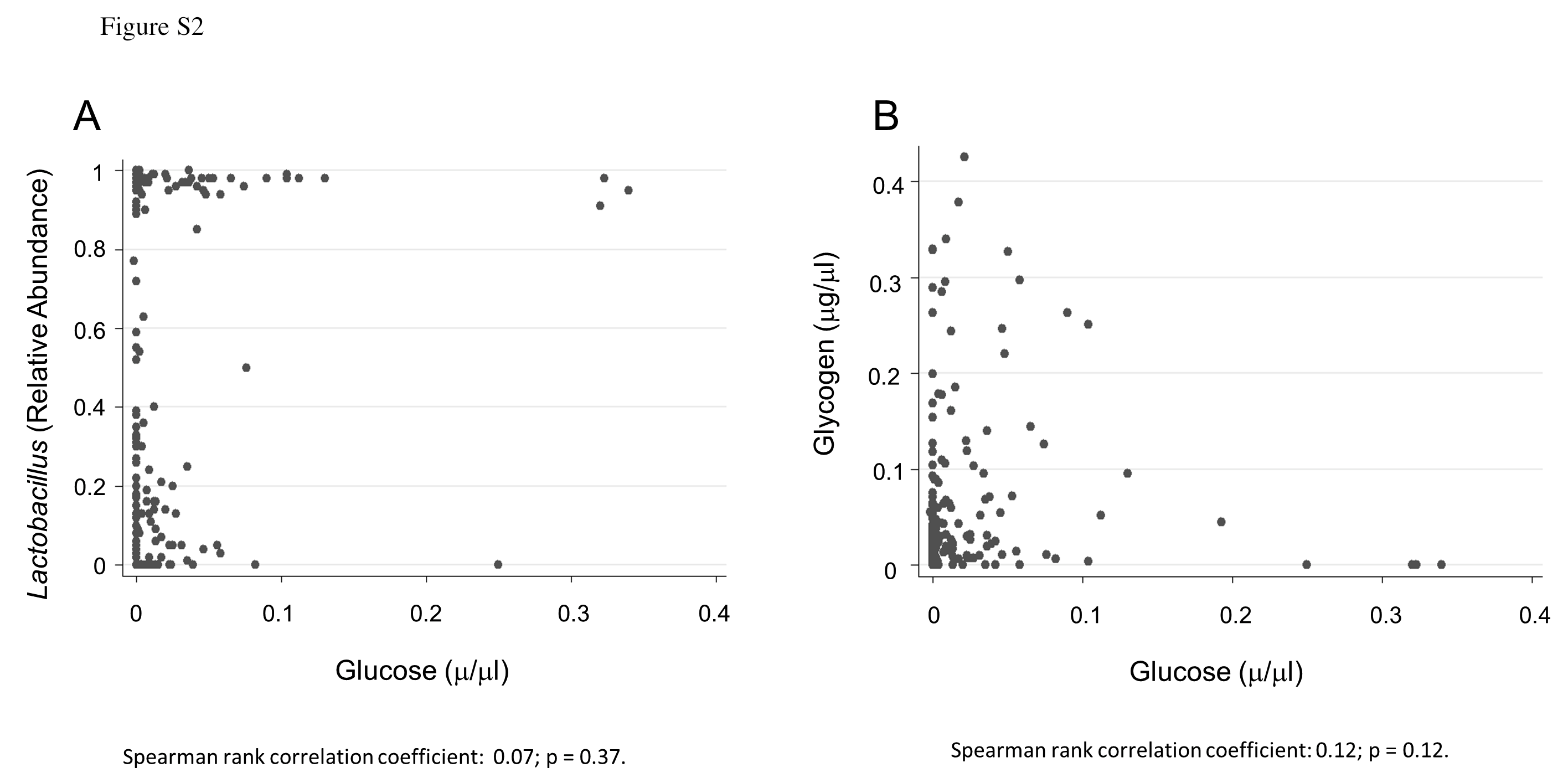

Supplement: Figure S2 — Distribution of Glucose by Lactobacillus and Glycogen. Glucose and glycogen levels were measured in vaginal fluids collected annually from 21 women over 8–11 years. The final analytic sample consisted of 177 observations with non-missing values for all variables. The distributions of Lactobacillus relative abundance (A) and glycogen (B) are summarized by glucose as a continuous variable. (TIFF) [file pone.0102467.s002.tiff]

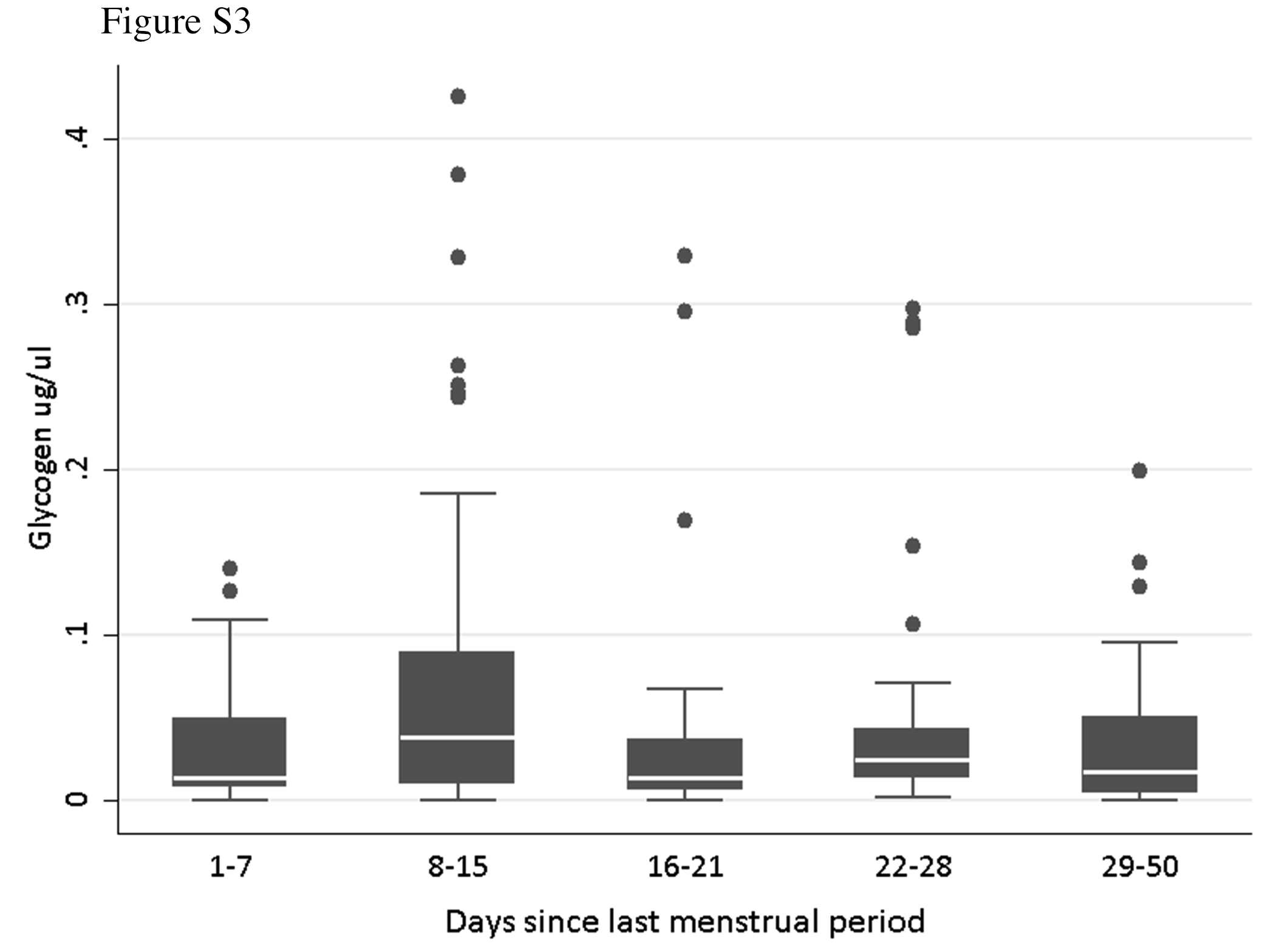

Supplement: Figure S3 — Glycogen Concentration by Time Since Last Menstrual Period (LMP). Shaded portions represent interquartile range, horizontal bars represent medians, and whiskers represent 95% confidence intervals. N = 149, excludes 6 visits where menopause or pregnancy was reported and 19 visits where LMP was >50 days. (TIFF) [file pone.0102467.s003.tiff]

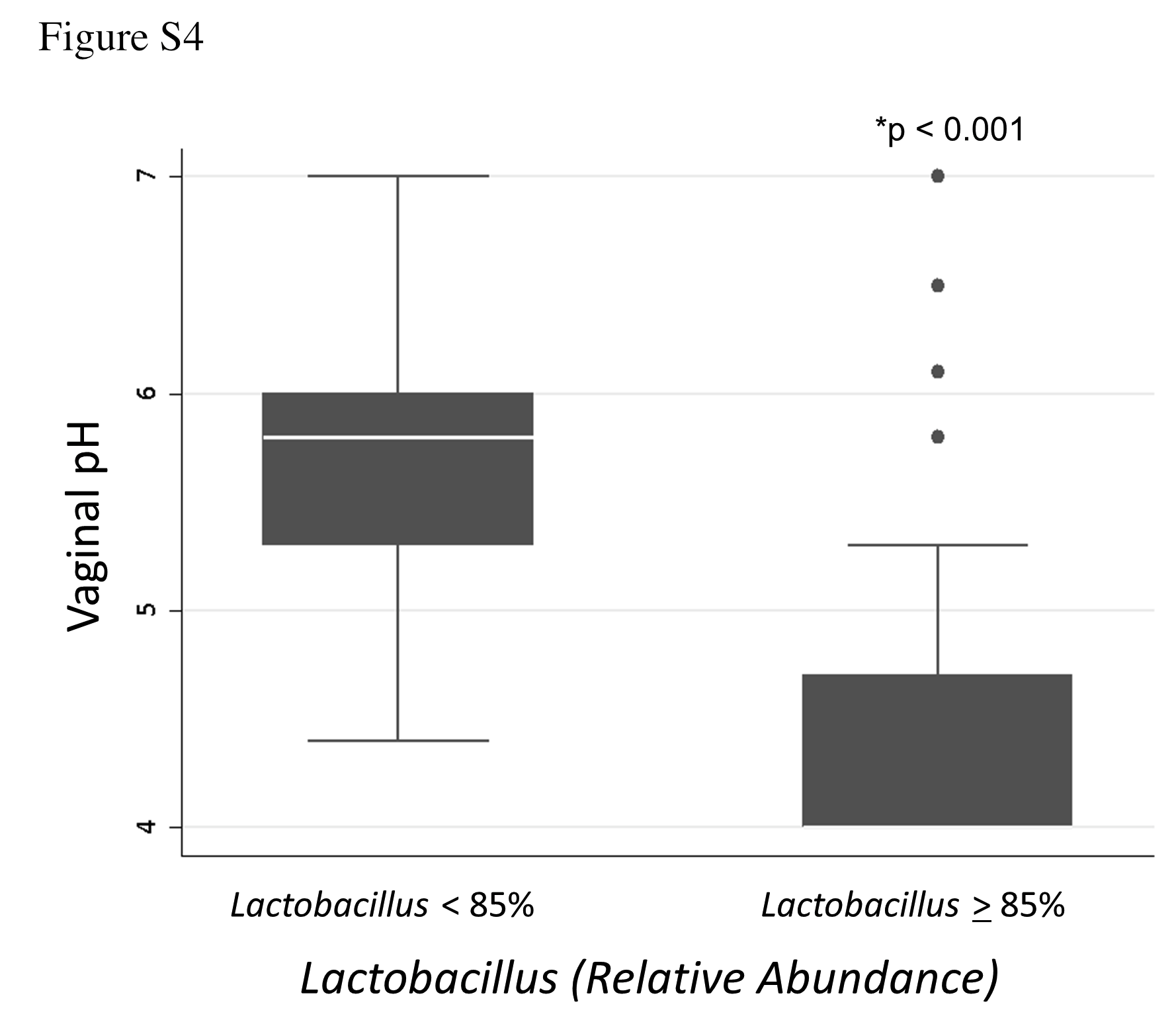

Supplement: Figure S4 — pH by Lactobacillus Relative Abundance ≥85 vs. <85%. (TIFF) [file pone.0102467.s004.tiff]
